# Social vulnerability from a social ecology perspective: a cohort study of older adults from the National Population Health Survey of Canada

**DOI:** 10.1186/1471-2318-14-90

**Published:** 2014-08-16

**Authors:** Melissa K Andrew, Janice M Keefe

**Affiliations:** 1 Division of Geriatric Medicine, Dalhousie University, Halifax, Nova Scotia, Canada; 2 Family Studies and Gerontology, Mount Saint Vincent University, Halifax, Nova Scotia, Canada

**Keywords:** Social vulnerability, Social ecology, Social isolation, Frailty, Frail elderly, Survival, National Population Health Survey, Canada

## Abstract

**Background:**

Numerous social factors, generally studied in isolation, have been associated with older adults’ health. Even so, older people’s social circumstances are complex and an approach which embraces this complexity is desirable. Here we investigate many social factors in relation to one another and to survival among older adults using a social ecology perspective to measure social vulnerability among older adults.

**Methods:**

2740 adults aged 65 and older were followed for ten years in the Canadian National Population Health Survey (NPHS). Twenty-three individual-level social variables were drawn from the 1994 NPHS and five Enumeration Area (EA)-level variables were abstracted from the 1996 Canadian Census using postal code linkage. Principal Component Analysis (PCA) was used to identify dimensions of social vulnerability. All social variables were summed to create a social vulnerability index which was studied in relation to ten-year mortality.

**Results:**

The PCA was limited by low variance (47%) explained by emergent factors. Seven dimensions of social vulnerability emerged in the most robust, yet limited, model: social support, engagement, living situation, self-esteem, sense of control, relations with others and contextual socio-economic status. These dimensions showed complex inter-relationships and were situated within a social ecology framework, considering spheres of influence from the individual through to group, neighbourhood and broader societal levels. Adjusting for age, sex, and frailty, increasing social vulnerability measured using the cumulative social vulnerability index was associated with increased risk of mortality over ten years in a Cox regression model (HR 1.04, 95% CI:1.01-1.07, p = 0.01).

**Conclusions:**

Social vulnerability has important independent influence on older adults’ health though relationships between contributing variables are complex and do not lend themselves well to fragmentation into a small number of discrete factors. A social ecology perspective provides a candidate framework for further study of social vulnerability among older adults.

## Background

### Social factors and older age: What is social vulnerability?

This paper aims to develop this concept of social vulnerability in old age and to situate it in the context of a human ecology theoretical framework. We then apply a theoretical model based on an ecological perspective of social vulnerability to an analysis of data from a longitudinal health survey of older Canadians.

“Social vulnerability” can be broadly understood as the degree to which a person’s overall social situation leaves them susceptible to health problems, where “health problems” are broadly construed to include physical, mental, psychological and functional problems [1-5]. Considered in the inverse, social reserve would be the degree of resilience that a well-connected and supportive social situation might impart. Though potentially relevant for people of all ages, social vulnerability is particularly important for older persons for a number of reasons. Firstly, there is growing evidence (briefly reviewed below) linking social circumstances in older age to health outcomes. Secondly, current policy contexts promote aging in place and care in the community despite the potential for dwindling social networks as one ages (due to frailty and death of friends and family members) and greater difficulty participating in social activities (due to health and functional challenges) [1,6].

### Social factors and health

Many social factors, including socioeconomic status (SES), deprivation, social support, social isolation or exclusion, social networks, social engagement, mastery and sense of control over life circumstances, social capital, and social cohesion have the potential to influence older adults’ health [1]. The literature in the field is substantial and expanding; key findings relating to social influences on older adults’ health are reviewed here.

SES, when considered broadly to include such factors as educational attainment, occupation and income, may influence older adults’ health in different ways; material deprivation, education-related health behaviours, and social/occupational status are three key proposed mechanisms [7]. For example, lower SES has been shown to predict cognitive decline independent of biomedical comorbidity [8]. Lower SES and living alone have been associated with increased risk of falls [9], and lower SES has also been identified as a predictor of frailty [10]. Subjective income adequacy was strongly associated with self-rated health in a study of community-dwelling Finnish older adults [11]. Self-perceived income adequacy was found to be a robust measure of SES in the Survey of Health, Ageing and Retirement in Europe, with the caveat that the oldest old (age 80+) may tend to underestimate their degree of financial difficulties [12]. The Whitehall studies of British civil servants identified a survival gradient across the occupational hierarchy that was closely correlated with SES [13]. Differences in health across the occupational hierarchy have also been linked to social status and occupational mastery/empowerment in what Marmot has described as a “status syndrome” [14].

SES is a property of individuals, but aggregate measures such as average neighbourhood income/deprivation, educational attainment and unemployment rates are useful for describing the social contexts in which people live and to allow study of so-called contextual effects on health. For example, neighbourhood-level SES has been associated with older adults’ cognitive function even once differences in individual SES are taken into account [15]. Neighbourhood-level deprivation has also been associated with incident mobility impairment and slow gait speed independent of individual SES and health status [16].

Availability of social supports has been associated with improved survival among older adults [17]. Emotional social support has also been identified as a predictor of better cognitive function [18]. Lack of social support has been identified as a risk factor for frailty [10] and care home placement [19]. Richer social networks have been associated with improved survival [20-22], lower incidence of dementia [23], reduced functional impairment [24], and delayed onset of physical disability among older adults [25]. Higher life satisfaction with social circumstances was associated with less depressive symptoms, lower disability and better cognition in the Manitoba Study of Health and Aging [26]. Social disengagement among older adults has been associated with incident cognitive decline [27] and increased disability [28].

Social capital is also important for health. Social capital can be understood to be a property of the links between people and communities [29-31]; for example Putnam has described social capital as “the features in our community life that make us more productive – a high level of engagement, trust, and reciprocity” (p.4) [32]. As an example of its impact on health, high social capital, defined by high levels of trust and volunteerism in communities, has been linked with reduced mortality [33,34]. Among older adults, high social capital (including engagement in group activities and trust in others) has been associated with higher levels of function and better self-assessed health [24]. Social capital has also been highlighted as an important issue for Public Health [33-36].

In addition to the associations with health outcomes reviewed above, social factors show important associations with mortality. For example, higher levels of perceived social support and social interaction have been associated with reduced mortality in community-dwelling older adults [17]. In the Alameda County study, participants with stronger social networks lad reduced mortality over nine years [20]. Seventeen-year follow-up data from the same study found that social conectedness predicted better survival at all ages, including those aged 70 and older [21]. Late-life social engagement has been associated with improved survival [37]. The Whitehall studies identified an impressive gradient in survival across levels in the occupational hierarchy; this gradient persisted after retirement among 70-89 year olds [13,14]. Ecological (collective-level) analyses using multilevel modeling have also linked high social capital, defined by high trust and membership in voluntary associations, with reduced mortality at state and neighbourhood levels in the United States [33,34].

As these examples illustrate, social influences on older adults’ health are both broad and diverse. Given this complexity, they also have the potential to merge and interact in complex and possibly unforeseen ways to create the “big picture” of social environments and circumstances in which individuals and communities live and exist. Despite this (and perhaps because of it, where simplicity has been seen as desirable), individual social factors have tended to be studied in isolation. While this adds to our understanding of which factors independently influence health outcomes, the big picture may be compromised by this artificial fragmentation. Oversimplification also brings with it the risk of misclassification. For example, in a study of the health impact of living alone, should an older person who is truly isolated with little support from family and friends be assigned the same risk state as one who compensates well with rich social networks and community engagement?

The level of influence at which the various social factors act and are measured is also important. Some social factors which contribute to overall social vulnerability are properties of individuals (such as educational attainment and income). Others are relevant at the group level, inhabiting a spectrum from the close family unit (*e.g.* marital status, living situation and family caregiving) through to wider peer groups (*e.g.* engagement in group activities), neighbourhood influences (*e.g.* neighbourhood deprivation), and the social cohesion of societies [1].

The diversity in social factors that are important for health, and the fact that they are relevant across the individual-to-group continuum, underscores the need for an integrated and comprehensive perspective of social influences on health. The main aim of this paper is therefore to explore the construct of social vulnerability and to present a conceptual framework which captures its relational dimensions.

Health and functional status are clearly important to any consideration of social vulnerability; the comprehensive construct of frailty is useful in this regard. There are many possible views and definitions of frailty [38-40]. While there is some controversy on the subject in the literature, each definition commonly considers frailty in terms of vulnerability. Some view frailty as a purely physical phenomenon; the frailty Phenotype defines frailty in terms of five characteristics (weakness, weight loss, exhaustion, inactivity, and slow walking speed; those with 0 phenotypic criteria are said to be non-frail, those with 1-2 are pre-frail and those with 3 or more are frail) [41-43]. Here, frailty is understood more broadly using another widely-used conceptualization in which frailty is understood as a state of susceptibility comprising illnesses, symptoms, and functional impairments which we have operationalized using a deficit accumulation approach; the number of problems that an individual has are summed to create a frailty index measure [43-45].

### Theoretical perspective

As we have seen, social vulnerability can be considered at various levels of influence from individual to close family, wider network, and societal context. A framework that explicitly considers these different levels of influence is therefore desirable. The human ecology perspective, originally proposed by Bronfenbrenner (1979), offers a conceptual frame which captures the interdependence of social factors and the contextual circumstances [46] that may seen as contributing to and/or mitigating social vulnerability. Bronfenbrenner (1979) described a system of nested interconnected layers of influence from the individual (molar) level through the “dyad, role, setting, social network, institution, subculture, and culture” (p. 8), and argued that this explicit consideration of the individual within micro- and macro-systems allows basic science and public policy to be reciprocally integrated (rather than having a one-way informing of policy by basic science) [47].

A criticism of the ecological perspective claims that it is a static and rigid descriptive model that is not sufficiently responsive to change over time [48]. If this were true, this would pose a problem for the conceptualization of social vulnerability, which is inherently dynamic and subject to changes in circumstances over both short term (*e.g.* death of a spouse or caregiver or sudden changes in an individual’s need for support that may or may not be met within their support network) and long term (*e.g.* gradual weakening of a social network, gradual declines in ability to engage in peer social groups). Bronfenbrenner later addressed this criticism by adding chronosystems, the dimension of time, to capture the effects of change and continuities. Chronosystems are typically conceived as life transitions - an important thrust that intersects with other systems. We agree that the ecological framework is best understood as a dynamic model. Changes can occurs over time and the model can be modified through directed intervention aimed at mitigating/reducing social vulnerability.

Various mechanisms have been proposed to explain how social factors might affect health; as these are active across various levels of influence, the social ecology perspective provides a useful framework for their consideration. Broadly speaking, these include four main groups. Notably, no single mechanism explains all of the observed variance, which once again highlights the complexity involved. *Physiological factors* clearly play a role. Chronic and sustained stress responses exert powerful effects on health through complex hormonal regulatory systems including activation of hormonal axes and effects on immune function [49]. *Behavioural factors* are also at play. Health-related behaviours such as diet, smoking, exercise and substance use are associated with social conditions (*e.g.*, SES and related opportunities, norms within social networks and communities) have important influences on health [50]. There are also clear *material influences.* SES and social support networks clearly affect access to goods and services. This access accrues in three broad ways: through financial resources (“what you have”), social status (“who you are”), and social contacts (“who you know”) [1]. Finally, *psychological factors* also play an important role. Self-efficacy and adaptive coping strategies are important for health. For example, low self-efficacy (having low confidence in one’s abilities) is associated with fear of falling, with important functional and mobility ramifications for older people [51] and also predicts functional decline in older people with impaired physical performance [28]. These factors have important influences at various levels from the individual to families, wider peer groups, institutions and societies, lending support to the usefulness of an ecological frame of reference.

Given our explicit focus on social factors from both individual and neighbourhood levels of analysis within this framework, we refer to it hereafter as a social ecology perspective. In doing so, we aim to acknowledge the agency of older persons themselves in the dynamic in play among the different layers of individual, family/peer group, community and society [48]. Conceptualizing social vulnerability within a social ecology framework, this study constructs and populates an ecological model of social vulnerability with empirical data. We then use this theoretical framework to discuss potential interventions at the various levels of influence (from individual to societal) that may be helpful in efforts to reduce social vulnerability among older people.

## Methods

### Sample

The National Population Health Survey (NPHS) is a longitudinal panel survey of Canadian residents of all ages administered by Statistics Canada. The sample was stratified according to geographic and socio-economic characteristics and clustered by Census Enumeration Area. Residents of aboriginal Reserves, Canadian Forces Bases, and some remote areas of Northern Canada were excluded from the NPHS. The survey was based on self-report or proxy-respondent interviews, and no clinical measures or biological samples were collected. The baseline data collection was in 1994, and the same sample was followed for ten years to determine survival. The response rate for all ages in the baseline cycle was 83.6%. 2740 participants in the NPHS panel sample were aged 65 or older at baseline in 1994 and were followed for 10 years; these individuals comprised the sample for this analysis. Ten-year survival status was determined through linkage to the Canadian Vital Statistics Database and was available for all of these 2740 participants. Analyses were weighted in order to account for the NPHS design and sampling methodology [52,53].

### Measures

#### *Individual-level variables*

Self-report variables pertaining to social factors with plausible health associations based on literature reviews were identified in the NPHS dataset [1,2]. Items were selected based on face validity with the aim of including as many diverse social factors as possible, given the aim of capturing complexity. We aimed to include as many items as possible from the NPHS dataset relating to socioeconomic circumstances and well as social support, engagement in community life, relationships with others and how participants subjectively perceived their social circumstances. Twenty-three individual-level social variables (plus five neighbourhood-level variables as described below for a total of 28 social variables) were indentified and included in our analyses (Table 1). Each variable was coded in terms of potential social “deficits” such that respondents were assigned a score of 0 if the deficit was absent and 1 if it was endorsed, with intermediate values applied in the case of ordered response categories [2]. For example, an individual scored 1 on the “living alone” deficit if he/she reported living alone, and 0 if he/she did not. On the “how often do you participate in group activities” item, which had five response categories, possible scores were 0 if the answer was “at least once a week”, 0.25 for “at least once a month”, 0.5 for “at least 3 or 4 times a year”, 0.75 for “at least once a year” and 1 for “not at all”. In this way, vulnerability on each item was mapped to the 0-1 interval and the scores were summed and divided by the total number of deficits considered to create a summary social vulnerability index. Emergent factors from a Principal Component Analysis (PCA) were identified as domains of social vulnerability. The 0-1 scores for the social variables that loaded onto each of these emergent factors were summed for each NPHS participant to generate a measure of his or her vulnerability for each domain.

**Table 1:** Principal component analysis with seven emergent factors

**7 factors**	**Rotated Component Matrix(a)**						
	**Component**						
	**1**	**2**	**3**	**4**	**5**	**6**	**7**
	**Engagement**	**Contextual SES**	**Social support**	**Living situation**	**Self-esteem**	**Sense of control**	**Relations w/ others**
Frequency of group engagement	.927*	-.037	-.022	-.032	.021	.006	.063
No group engagement	.917*	-.029	-.018	-.003	-.006	.013	.050
Attending religious services	.471*	.065	.141	.056	-.035	-.018	.138
Physical leisure activities	.311*	-.223	.043	.151	.246	.032	-.056
EA income	.004	.783*	-.010	-.106	.014	-.021	-.195
EA education	.000	.735*	-.022	.002	-.076	-.010	.264
EA inequality	.034	-.704*	.054	.001	-.046	-.029	-6.15E-005
EA unemployment	.015	.582*	.049	.007	.010	-.059	-.147
Support: advice	.055	-.012	.736*	.039	-.065	.109	.011
Support: help in a crisis	.019	-.019	.713*	-.061	.008	.025	-.014
Support: someone to confide in	.061	-.027	.680*	-.107	.001	.136	.006
Support: someone to make you feel loved	.033	-.006	.583*	.196	.119	.020	.002
Frequency of contact with relatives	-.017	.030	.372*	.151	.039	-.121	.216
Lives alone	.018	-.035	.076	.940*	-.008	.046	-.036
Marital status	.054	-.051	.057	.937*	.013	.053	-.016
Worth equal to others	-.012	-.018	.022	.025	.846*	-.016	.032
Positive attitude towards self	.014	.027	.046	-.032	.841*	.086	.082
Too much expected of you by others	-.122	.007	.016	-.030	-.059	.566*	.026
Want to move but cannot	.050	-.001	.036	-.008	.074	.560*	.150
Not enough money to buy the things you need	.067	-.083	.039	.004	.006	.524*	-.101
Little control over things that happen	.078	-.025	.046	-.015	.263	.488*	-.207
How often have people let you down	-.011	.045	.115	.127	-.003	.462*	.176
Noisy/polluted neighbourhood	-.004	-.064	-.106	.044	-.087	.392*	.360
Frequency of contact with neighbours	.092	.029	.147	-.053	.120	.067	.555*
EA caregiving for seniors	.001	.149	.025	-.065	.128	.006	-.500*
Frequency of contact with friends	.274	-.012	.241	-.195	.091	.049	.405*
Education	.262	-.344	.017	-.069	.166	.148	-.386
Ability to speak English or French	.070	.003	-.012	-.026	.084	.047	.178

Each variable’s factor loading is indicated by *.

Extraction Method: Principal Component Analysis. Rotation Method: Varimax with Kaiser Normalization. Rotation converged in 6 iterations.

EA = Enumeration Area.

Individual-level covariates considered were age, sex, educational attainment and frailty. Age was measured in years, with the entire sample being aged 65 or older. Sex was included with the aim of investigating and accounting for gender differences in social vulnerability and survival. Previous work with the social vulnerability index has found that women are more socially vulnerable than men, though women have greater survival [2]. Educational attainment was measured using the Statistics Canada four-level derived variable (less than secondary schooling, secondary school completed, some post-secondary education, and completed post-secondary education). Although education was included among the social variables in the PCA, it did not load onto any one domain and was not included in the composite social vulnerability index. Due to the *a priori* relevance of age, sex, educational attainment and frailty, all four were included as covariates in regression models.

The frailty index comprised 35 health deficits, including sensory and functional impairments, symptoms, and illnesses, with coding analogous to the social vulnerability index described above. As such, the frailty index is based on a count of health deficits, and represents the number of health-related problems that a person has as a proportion of the total number of deficits considered in the index. The NPHS frailty index has been previously validated, and is robustly predictive of mortality [44]. The frailty index approach has been applied in many settings, across multiple populations and countries, and demonstrates remarkably consistent properties. For example, frailty increases with age as deficits accumulate at an average rate of three percent per year, women have higher frailty than men at all ages (but show better survival at any given level of frailty), and frailty is more robustly predictive of mortality than chronological age [44,54]. There are also preserved and reproducible limits to how frail people can become [55]. The frailty index shows consistency and convergent validity with other measures of frailty and comorbidity, though has increased potential for predictive discrimination between grades of frailty [43,56,57]. The deficit accumulation approach employed by both the frailty index and the social vulnerability index reduces dimensionality and allows much information to be included in statistical models without having many separate parameters, which can otherwise lead to problems with multiple colliniarity and model instability.

#### *Neighbourhood-level variables*

Recognizing the need to include macro-level contextual variables, a series of socioeconomic and socio-demographic characteristics of neighbourhoods were constructed by postal code linkage of individual participants to their Enumeration Area (EA) of residence using the Canadian Census. Group level variables considered were aggregate income, education, income inequality, and unemployment. Given our focus on older adults, we also included an aggregate measure of caring for seniors. Linkage of individual participants in the NPHS to the aggregate variables describing their neighbourhoods was done using the Statistics Canada Postal Code Conversion File “plus” (PCCF+), which has been designed to rigorously assign postal codes to geographical areas units based on the best available data with built-in troubleshooting and detection of errors [58]. The 1996 census was chosen to be as closely contemporaneous to the time of data collection as possible. EAs (which have subsequently been renamed “Dissemination Areas” since the 2001 census) are the smallest units of census geography aggregation for which data are released, and include an average of 400-700 individuals [59]. Both the PCCF + and the raw census data were obtained through the Statistics Canada’s Data Liberation Initiative, which facilitates access to data for academic use [60]. Characteristics of the EAs were based on a 20 percent subsample of residents who completed a more detailed “long form” census questionnaire, and were downloaded using the online Census Data Analyser. For the purposes of the present analysis, each EA was assigned to a quintile for each of income, income inequality, education, unemployment, and caregiving for seniors based on the raw counts for each EA reported in the census.

*Income* quintiles were based on within-area comparisons rather than on national absolute values. This was done in order to take regional differences in income norms and cost of living into account. This variable has been previously developed and used for the Canadian Census, and was obtained from the author of the PCCF + [58]. Coding was such that 1 represented the lowest income quintile and 5 the highest. Income inequality quintiles were generated from the standard error of income within each EA. *Income inequality* was coded such that 1 was the most unequal and 5 the most equal. Income inequality was examined because of evidence from other studies that inequality, even within neighbourhoods, can have negative consequences for health [61,62]. The proportion of *unemployment* among census respondents aged 25 years and over was used to divide EAs into quintiles of unemployment from 1 (lowest unemployment) to 5 (highest). *Educational attainment* within the EA was calculated based on the proportion of adult respondents who reported having completed high school. Each EA was assigned a quintile of educational attainment coded from 1 (lowest proportion of high school completers) to 5 (highest).

The *caregiving for seniors* variable took into account both the number of adult respondents who reported having provided any regular unpaid care to an older person (as a proportion of the total adult population of the EA) and the proportion of the EA’s population that was over the age of 65. Each EA was assigned a weight in relation to the mean 65+ population for all EAs, such that EAs with a larger proportion of seniors would have a weight value >1, and those with fewer seniors as a proportion of their total population would have a weight <1. This allowed the proportion of respondents who reported themselves as care-providers to be adjusted for the ambient numbers of seniors by dividing by the senior population weight. This was done in order to ensure that the caregiving variable measured altruistic caregiving behaviour rather than just providing a measure of the relative numbers of seniors “available to be cared for” in each EA.

#### *Missing data*

Complete data for the social vulnerability variables was available at baseline for 2058 individuals; 682 were missing data for at least one social variable. Those who had missing social data were older (p = 0.01) and more frail (p = 0.05). Individuals with complete data for some but not all social vulnerability domains were included in as many of the analyses as possible. Because of this effort, the total numbers included in the analyses of each domain were slightly different. The total numbers with complete data for each covariate and domain were as follows: Age and Sex N = 2740, Educational attainment N = 2728, Frailty N = 2655, Engagement N = 2534, Contextual SES N = 2419, Social support N = 2529, Living situation N = 2740, Self-esteem N = 2512, Sense of control N = 2490, and Relations with others N = 2204.

#### *Statistical methods*

First, factor analysis was conducted using SPSS 15.0 in order to understand how vulnerability variables organize into specific domains. Factor loadings were calculated using Principal Component Analysis with Varimax rotation. Seven factors were specified based on examination of the scree plot, as there was diminishing contribution from additional factors after the first seven (for example, 14 factors would be required to explain 70% of the variance). Next, an exploratory analysis using descriptive and regression analyses were done using Stata 10.1, Associations of each dimension with age, sex, frailty, and education, were studied using linear regression models in order to obtain a more holistic view of the interrelationship of individual domains. Regression models rather than zero order correlations were used in order to allow adjustment for relevant covariates.

In order to link these results to health outcomes, we assessed the association between social vulnerability and all-cause mortality. In the summed social vulnerability index and mortality we aimed to re-assemble the domains of social vulnerability into a combined measure. Associations between social vulnerability index and survival were tested using logistic and Cox regression models. All regression models were adjusted for covariates with *a priori* significance (age, sex, frailty, and education). In order to ensure that the underlying assumptions of Cox regression were not violated, the proportionality of hazards assumption was tested graphically using Schoenfeld residuals. Proportional sampling weights were used to account for sample design [52].

#### *Ethics and data access*

The NPHS was conducted by Statistics Canada and was approved by the Statistics Canada ethics review process, with participants providing informed consent. For these analyses, NPHS datasets were accessed through an agreement with the Statistics Canada’s Atlantic Regional Data Centre, which obliged the author (MKA) to operate, for these purposes only, as a “deemed employee” of Statistics Canada. Statistics Canada officials reviewed the analyses to ensure that confidentiality had not been breached. Secondary analyses were approved by the Research Ethics Committee of the Capital District Health Authority, Halifax, Nova Scotia.

## Results

The mean age was 73.4 years (95% CI: 73.0-73.7), and 57 per cent of the sample were women. Educational attainment was low, with 54 per cent of the sample having attained less than secondary schooling. The mean frailty index score in this sample of community-dwelling older adults was 0.11 (95% CI: 0.10-0.11), which, according to published cut-offs of 0.2-0.25, is not frail [43,63] (Table 2). The distributions of variables included in the Social Vulnerability Index are summarized in Table 2 to indicate the burden of vulnerability attributable to each item. At 10 years, 1437 (52%) of the 2740 individuals in the sample had died.

**Table 2:** Description of sample (all results weighted to reflect sampling methodology) and of variables included in the Social Vulnerability Index

**Variable**	**Mean (95% CI) or %**
Age	73.4 (73.0-73.7)
Gender (female)	57%
Frailty index	0.11 (0.10-0.11)
Education	
Less than secondary school graduation	54.1%
Secondary school graduation	12.8%
Some post-secondary	16.3%
Post-secondary graduation	16.9%
Frequency of group engagement	0.68 (0.66-0.71)
No group engagement	0.58 (0.56-0.61)
Attending religious services	0.46 (0.44-0.49)
Physical leisure activities	0.67 (0.65-0.68)
EA income	0.56 (0.54-0.58)
EA education	0.52 (0.50-0.54)
EA inequality	0.49 (0.48-0.51)
EA unemployment	0.51 (0.49-0.52)
Support: advice	0.11 (0.09-0.13)
Support: help in a crisis	0.05 (0.04-0.06)
Support: someone to confide in	0.17 (0.15-0.19)
Support: someone to make you feel loved	0.03 (0.02-0.04)
Frequency of contact with relatives	0.15 (0.14-0.16)
Lives alone	0.31 (0.29-0.33)
Marital status	0.41 (0.39-0.43)
Worth equal to others	0.15 (0.14-0.16)
Positive attitude towards self	0.17 (0.16-0.17)
Too much expected of you by others	0.13 (0.12-0.15)
Want to move but cannot	0.10 (0.09-0.12)
Not enough money to buy the things you need	0.18 (0.16-0.20)
Little control over things that happen	0.37 (0.36-0.39)
How often have people let you down	0.25 (0.24-0.26)
Noisy/polluted neighbourhood	0.06 (0.05-0.08)
Frequency of contact with neighbours	0.29 (0.27-0.31)
EA caregiving for seniors	0.48 (0.46-0.50)
Frequency of contact with friends	0.26 (0.25-0.28)
Ability to speak English or French	0.04 (0.03-0.05)

EA = Enumeration Area.

**Figure 1:**
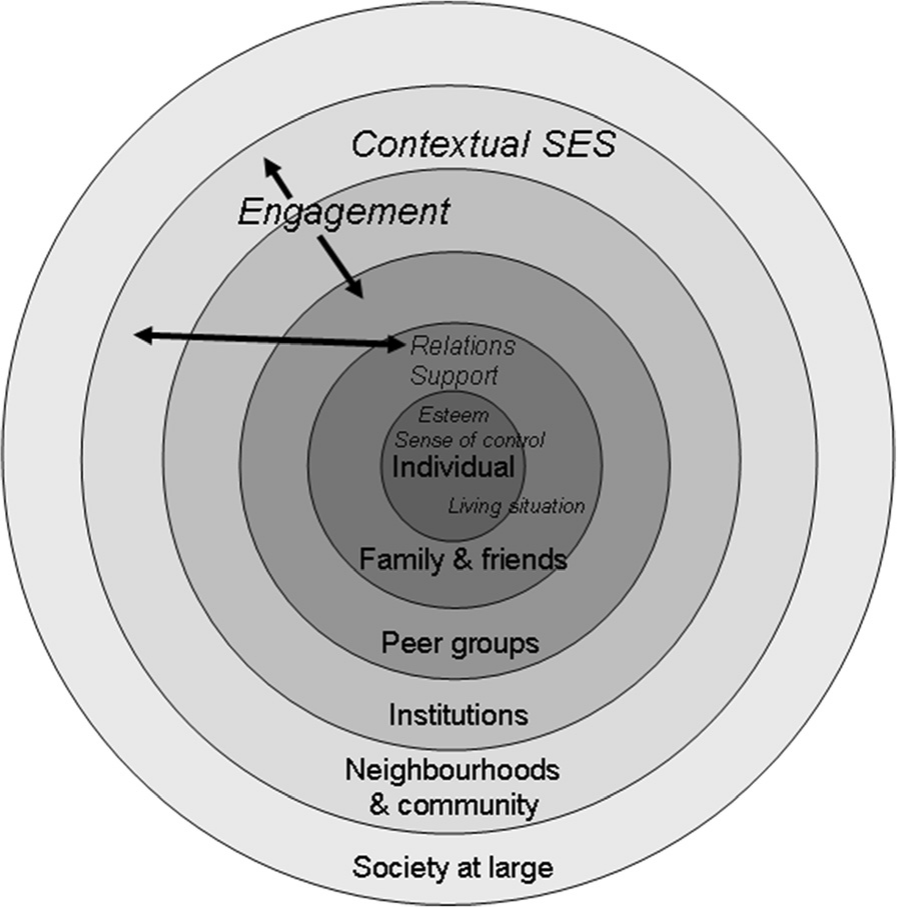
**Dimensions of social vulnerability situated within the ecological model of social vulnerability.** The seven emergent dimensions of social vulnerability (in italics) are situated within the ecological framework, which includes spheres of influence from the individual, to close family and friends, wider peer groups, institutions, community, and society.

Seven factors emerged from the Principal Component Analysis, explaining 47.0 per cent of the total variance (Table 1). As outlined in Table 1, the seven dimensions were self-esteem, sense of control, living situation, engagement in social activities, social support, relations with others, and contextual SES. Two variables, educational attainment and language (ability to speak English or French) did not load onto a single factor. Figure 1 shows the seven dimensions of social vulnerability situated within the social ecology framework, with the identified dimensions of social vulnerability located across spheres of influence from the individual, to close family and friends, wider peer groups, institutions, community, and society. Of note, some dimensions span spheres, whether adjacent (in the case of living situation, which incorporates individual as well as close family spheres) or more distant (as is the case for engagement with others, which is relevant for both the family and friends, as well as neighbourhood/community spheres).

Vulnerability in each dimension was explored in relation to the four covariates considered: age, sex, education, and frailty (Table 3). Women reported lower vulnerability in social engagement and social support, but were more vulnerable in terms of their living situation (living alone and/or being single or widowed) (p < 0.001). Vulnerability in living situation and in sense of control increased with increasing age (both p < 0.001). Higher levels of frailty were associated with more vulnerability in the engagement, sense of control, and self-esteem dimensions (all p < 0.001). Older adults with lower education reported more vulnerability in four of the domains: engagement, sense of control, contextual SES (all p < 0.001) and self-esteem (p = 0.01).

**Table 3:** Associations of the social vulnerability dimensions with covariates (gender, age, frailty, and education)

	**Engagement N = 2516**	**Contextual SES N = 2342**	**Support N = 2512**	**Living situation N = 2648**	**Esteem N = 2512**	**Control N = 2473**	**Relations N = 2188**
Female Gender	-0.13 (-0.25, -0.01)*	-0.05 (-0.13, 0.03)	-0.14 (-0.23, -0.05)**	0.52 (0.44, 0.60)***	-0.00 (-0.03, 0.03)	-0.06 (-0.16, 0.03)	-0.03 (-0.10, 0.05)
Age (increasing)	0.01 (-0.01, 0.02)	-0.00 (-0.01, 0.00)	-0.01 (-0.01, 0.00)	0.03 (0.02, 0.03)***	-0.00 (-0.00, 0.00)	-0.02 (-0.03, -0.01)***	0.00 (-0.00, 0.01)
Frailty (increasing)	1.37 (0.77, 1.96)***	0.35 (-0.03, 0.73)	0.05 (-0.29, 0.58)	-0.11 (-0.50,0.27)	0.39 (0.22, 0.55)***	2.22 (1.74, 2.71)***	0.22 (-0.18, 0.61)
Education (lower)	0.42 (0.26, 0.58)***	0.34 (0.25, 0.44)***	0.05 (-0.06, 0.15)	0.03 (-0.07, 0.13)	0.04 (0.01, 0.08)**	0.02 (0.09, 0.32)***	0.06 (-0.03, 0.14)

Statistically significant associations are indicated by *p < .05 **p < .01 ***p < .001. The Number of participants included in the regression model for each dimension is indicated.

Adjusting for age, sex, and frailty, increasing social vulnerability as measured using the cumulative social vulnerability index was associated with increased odds of mortality over ten years in a logistic regression model (OR 1.05, 95% CI: 1.00-1.10, p = 0.04). These results mean that for every additional social deficit, an individual’s odds of mortality increased by 5%. Similar results were obtained using Cox regression (HR 1.04, 95% CI:1.01-1.07, p = 0.01).

## Discussion

We identified seven dimensions of social vulnerability (self-esteem, sense of control, living situation, social support, engagement, relations with others, and neighbourhood SES) in a sample of 2740 older Canadians and situated them within a social ecology framework of social vulnerability. The low percentage of variance explained by the seven dimensions is a limitation of our study and reflects the challenge of parsing many very different contributing factors to overall social vulnerability (from self esteem to SES to social supports and engagement) into distinct domains. As a result, one important interpretation of our findings is that a deficit accumulation approach, or social vulnerability index, is more appropriate for the conceptualization and study of social vulnerability. Nevertheless, our attempt at factor analysis does illustrate that inter-relationships between the social variables that contribute to overall social vulnerability have complex inter-connections. The factor loadings are far from clean-cut and thus do not tell a simple tale – individual variables load onto different domains, again illustrating their complex interrelationships.

Exactly which factors should contribute to the construct of social vulnerability is debatable. We aimed to include as many social variables as possible, in order to create a rich and comprehensive measure reflecting, as well as possible, the complexity of older adults’ social circumstances. Since our aim was to be as holistic as possible, we included self-esteem and mastery, though it could be argued that these are psychological traits and not social factors. There is considerable evidence that one’s sense of control over life circumstances is an important contributor to both social status and health, and they have been identified as potential mechanisms for observed associations between social factors and health [14]. Inattention to the relationship between self-perception and interactions with others runs the risk of fragmenting understanding of the relationship between social factors and health status. Our finding that vulnerability in the self-esteem and sense of control domains was associated with vulnerability in other domains supports the inclusion of these domains in the comprehensive conceptual model that we propose. The loading of individual variables onto domains of social vulnerability in the PCA is also of course open to interpretation. For example, five variables loaded together onto a domain which we have called “social support”. Social support is a broad construct, which can mean different things in different settings. Here, our measures of social support are based on self-report, and are subjective. They describe support of a more emotional than instrumental nature: someone to confide in, make one feel loved, offer help in a crisis, and give advice, along with a measure of reported contact with relatives. If a different list of variables contributing to overall social vulnerability was used, the factor loadings onto domains might be different. This is one reason why we find the composite social vulnerability index to be a useful approach – it is less dependent which specific variables are included or excluded [2].

Exploratory analysis of associations between the seven identified dimensions of social vulnerability and the covariates age, sex, frailty, and education yielded some results that may help to clarify differences in social vulnerability as faced by members of different groups of older adults (*i.e.* the oldest old, women, those with low education, and those who are frail). Those with lower education reported lower engagement, lower self-esteem, lower sense of control, and had lower contextual SES (a measure which included neighbourhood educational attainment). This is consistent with existing literature concerning the importance of education across many social measures [64,65]. The more frail people reported lower self-esteem and lower sense of control, which support assertions of the global importance of frailty and function on peoples’ self-conceptualization [38]. This finding is also consistent with existing literature reporting a link between functional disability, comorbidity, and low mastery [66]. It is not surprising that the more frail people had lower levels of engagement in social activities. Given the functional and illness elements within the definition of frailty, those who are ill and functionally impaired would likely have a more difficult time travelling to and participating in such activities. Taking a bigger picture view, frailty and social vulnerability are likely to have a reciprocal relationship, though their correlation is only moderate [2].

In exploratory analyses, increasing age was associated with more vulnerability in sense of control and living situation (i.e. people were more likely to be widowed and/or living alone at older ages). Women had higher reported engagement and social support but were more vulnerable in their living situation (*i.e.* living alone and/or being single or widowed). Previous analyses using the social vulnerability index have found that women are more socially vulnerable than men, and that in both sexes, higher social vulnerability predicts mortality independent of age and frailty [2]. The present analysis of the constituent domains of social vulnerability helps to clarify this point. Although women may have higher social support and engagement, their greater vulnerability in living situation (chiefly widowhood and living alone) seems to drive their higher levels of overall social vulnerability. This finding suggests that sex differences in social vulnerability profiles warrant further study.

Our findings should be interpreted with caution. The factor analysis was limited, as discussed above, and domains which contribute to social vulnerability merit further study and replication. All measures were based on self-report in a large social survey. It is possible that this subjective perception of social vulnerability may differ from an assessment that is based on more objective measures. However, it is also conceivable that a person’s self-perception of their social circumstances may contribute to their social vulnerability in important ways that are independent of the objective set of circumstances. For example, self-perceived income adequacy has been found to be a robust measure of SES in older adults [12]. Only all-cause mortality is known in the NPHS, so cause of death is not possible to examine, and data on other important health outcomes are not available. Increases in vulnerability with age were noted, though with the study design (a single cohort of older adults aged 65+) it is not possible to determine the relative contribution of age *vs*. cohort effects. Additionally, although the ecological model of social vulnerability considers spheres of influence at group (family, peer) and community (institutions, neighbourhoods, and society) levels, all measures were based on individual-level data. Even the census measures (relative educational attainment, income, income inequality, and community caregiving for seniors), while not originating directly from the individual NPHS participants, were aggregated at the EA level from individual responses to the 1996 census. There is debate surrounding the level, from individual to communal, at which some elements of the social context are relevant, and as such, how they can be measured [6,67,68]. Ideally some group-level measures would have usefully expanded the model, though these are difficult to obtain. Some suggestions in the literature include assessments of neighbourhood orderliness (*e.g.* lack of graffiti and litter, few abandoned buildings), civic engagement (*e.g.* rates of voting and newspaper subscriptions) and trust (*e.g.* gas stations not requiring payment prior to gasoline pumping) but these measures were not available here [31]. The paucity of true ecological measures represents an identified challenge for the study of social environments and health [67,68]. In a related issue, the true societal level was not well represented in our data. Ideally, including an analysis of regional policies might strengthen discussion in regard to the societal sphere of influence.

Missing data, due to both non-participation and item non-response, present another set of limitations. Individuals missing the social vulnerability variables were likely to be missing several of the individual items. This could be due to refusal to answer sections of questions about one’s private feelings and social circumstances or to the problem of proxy respondents not knowing how to answer personal questions about their loved one. As such, each domain had a slightly different “N” in the statistical analyses which we have reported in the Methods section. One could take the approach of imputing missing data, but if multiple items are missing this could itself compromise estimates. Respondents with missing data were likely to be older and more frail. Self-report data of this nature in older adults is commonly missing for those in whom proxy respondent is used. This “silence by proxy” is therefore a limitation of studying self-reported issues in frail older people, as the frailest (and arguably those for whom assessment and intervention for social vulnerability may be of greatest import) may be unable to answer for themselves [6,24]. Given this, our estimates of the importance of social vulnerability may be conservative. In particular, associations with mortality may well be conservative given that social vulnerability tends to increase with age and frailty [2]. Even so, missing social data amongst the oldest and frailest, who are arguably among the most vulnerable groups in society, unfortunately limits our ability to generalize our results to state that social vulnerability independently predicts mortality in the oldest and frailest community dwellers. Ideally this will be a focus of future study, though challenges abound. With regard to the issue of survey non-participation, it is also reasonable to assume that these factors may be associated with non-participation in the NPHS survey as a whole (as part of the overall response rate of 83.6% in the 1994 cycle interview), although these data are not published by Statistics Canada. Also relating to generalizability, the findings presented here apply to older Canadians included in the NPHS sampling frame (for example those living on aboriginal reservations, in remote Northern communities and on military bases were excluded from sampling). They may well be specific to a Canadian context, in as much as countries’ health and social systems differ. Further study of social vulnerability in international settings is warranted.

Another limitation to our approach is that the seven dimensions of social vulnerability identified here accounted for only 47% of total variance. One potential contributing factor to the low variance explained is that the social variables considered here were not taken from a preexisting discrete scale – as we have discussed above, we aimed to be as comprehensive as possible in including variables which could relate to social vulnerability rather than limiting our analyses to existing scales of single concepts (e.g. only social support, only engagement, only SES…) within the NPHS. In any factor analysis, the operator can choose to set certain parameters, including the number of factors to be discovered. In the course of our analyses, we thought carefully about the number of factors to consider, and repeated iterations of the factor analysis specifying different numbers of factors. In the end, the model with seven factors was the most stable (*e.g.* converging in a reasonable number of iterations, identifying factors which make sense in the context of the social ecology theoretical framework and maximizing the explained variance), and also captured a coherent picture of dimensions of social vulnerability in which the loading of individual variables made sense. The relatively low percentage of variance suggests that including additional latent factors might improve the descriptive power of the approach. Taking this to extremes, a model that includes 28 factors would by definition explain 100% of the variance; 14 factors would be required to explain 70% of the variance. This observation supports the idea that social vulnerability is a global construct that may be best considered as a whole, and that it may not lend itself well to being parsed into a small number of defined bits for separate analysis of individual social factors, or dimensions, in isolation. The importance of overall social vulnerability is also supported by our finding (here and elsewhere) that the full social vulnerability index, which combines all of the social variables, is associated with important health outcomes [2-5]. The index approach also has the benefit of allowing mathematical modeling to be used along with traditional statistical methods. On the other hand, it is useful to think about domains of social vulnerability as a starting point for considering social vulnerability within a theoretical framework, and for considering how important demographic variables are associated with different aspects of overall social vulnerability, which is why we have undertaken factor analysis and the associated exploratory analyses here. Testing this framework in other populations and datasets will be important in order to investigate whether the dimensions of social vulnerability that emerge are consistent or different between studies and settings.

The analysis presented here builds on and complements in important ways our prior work on the social vulnerability index, which was more empirically and clinically grounded [2]. Here we introduce the ecological perspective as a conceptual framework for considering social vulnerability, which situates the complexity of older people’s social circumstances within multiple spheres of influence. The use of PCA allows for consideration of domains of vulnerability, which allows exploration of some of the questions that have arisen in the course of our work – *e.g.* gender differences in social vulnerability, how domains are inter-related, and how they are associated with important covariates such as age and frailty. Consideration of domains contributing to social vulnerability may also allow for identification of contributors to social vulnerability that may be suitable targets for interventions on both clinical and policy levels.

Some may question why it is relevant to add together the individual domains to create the summative social vulnerability index. Interestingly, work with index variables done by our group and others has shown that adding together many variables, even if each is not individually associated or is only weakly associated with an outcome of interest, can yield important predictive validity. In this way, an index of nontraditional risk factors combine to predict dementia risk [69], and cumulative accumulation of deficits predicts changes in health status and mortality [57,70].

Our finding that the global construct of social vulnerability was associated with mortality is consistent with previously published findings using the social vulnerability index in the NPHS and Canadian Study of Health and Aging [2,4]. It also highlights a challenge for policy making, in that it may be difficult to appropriately design and target interventions to address overall social vulnerability. Few strategies have been proven to improve the social factors that contribute to social vulnerability, and few social interventions have been rigorously studied in relation to health outcomes to date. Further research is needed in this area. The ecological perspective presented here provides a framework for considering potential policy interventions.

Such interventions could be targeted at each of the various levels of influence within the ecological model. For example, at the individual level, policies aimed at improving educational opportunities, pension support, and interventions to address frailty and disability could be useful. Policies to support caregivers might help to enhance social support, and facilitation of opportunities to interact with friends and peers, though provisions of common space in living facilities and support of community groups, could improve opportunities for meaningful social engagement [71]. Age-friendliness of communities is also important, and there is potential to mitigate the extent and effects of social vulnerability by ensuring that communities are accessible to people of all ages and abilities [72].

Of note, there is considerable overlap in the spheres of influence within the ecological model that many of these interventions could influence, which again highlights the inter-relationship and inter-connectedness of the different levels from individual to society. For example, caregiving and support within a family or peer group relies on the individual’s willingness to receive support, and also relates to norms of caregiving behaviour within the broader community. Policies that seek to support caregivers could thus be facilitated by, or encounter barriers, at numerous levels of the ecological model. From a policy perspective, our findings also raise the question of whether it is better to implement a number of narrowly focused policies, each aimed at a different issue in a different part of the ecological model, or a single “omnipolicy” which is comprehensive and complex.

## Conclusions

We have proposed a social ecology perspective on social vulnerability which may serve as a useful framework for future studies and interventions addressing this important issue. We argue that considering social factors that influence health within an integrated and comprehensive framework rather than one at a time will allow for a richer understanding of how social environments affect older people’s health. Additional study in other samples and settings, ideally with different types of methodologies and measures, will hopefully further our understanding of social vulnerability among older people.

## Abbreviations

EA: Enumeration area; NPHS: National Population Health Survey; PCA: Principal component analysis; SES: Socioeconomic status.

## Competing interests

The authors declare that they have no competing interests.

## Authors’ contributions

MKA and JK conceived of and designed the study. MKA performed the analyses and wrote the initial draft of the manuscript. Both authors revised the manuscript, and have approved the final version.

## Pre-publication history

The pre-publication history for this paper can be accessed here:

http://www.biomedcentral.com/1471-2318/14/90/prepub

## References

[B1] Andrew MK Rockwood K, Fillit H, Woodhouse K Social vulnerability in old age Brocklehurst's Textbook of Geriatrics and Gerontology 2010 7 Philadelphia: Saunders Elsevier 198 204

[B2] Andrew MK Mitnitski A Rockwood K Social vulnerability, frailty, and mortality in elderly people PLoS One 2008 14 5 e2232 18493324 10.1371/journal.pone.0002232 PMC2375054

[B3] Andrew M Fisk JD Rockwood K Social vulnerability and prefrontal cortical function in elderly people: a report from the Canadian Study of Health and Aging Int Psychogeriatr 2011 14 1 9 10.1017/S1041610210001195 20699047

[B4] Andrew M Mitnitski A Kirkland SA Rockwood K The impact of social vulnerability on the survival of the fittest older adults Age Ageing 2012 doi:10.1093/ageing/afr176 10.1093/ageing/afr176 22287038

[B5] Andrew MK Rockwood K Social vulnerability predicts cognitive decline in a prospective cohort of older Canadians Alzheimers Dementia 2010 14 4 319 325 10.1016/j.jalz.2009.11.001 20630414

[B6] Andrew MK Le capital social et la santé des personnes âgées Retraite et Société 2005 14 129 143

[B7] Grundy E Holt G The socioeconomic status of older adults: how should we measure it in studies of health inequalities? J Epidemiol Community Health 2001 14 12 895 904 11707484 10.1136/jech.55.12.895 PMC1731799

[B8] Koster A Penninx BW Bosma H Kempen GI Newman AB Rubin SM Satterfield S Atkinson HH Ayonayon HN Rosano C Yaffe K Harris TB Rooks RN Van Eijk JT Kritchevsky SB Socioeconomic differences in cognitive decline and the role of biomedical factors Ann Epidemiol 2005 14 8 564 571 15922627 10.1016/j.annepidem.2005.02.008

[B9] Gill T Taylor AW Pengelly A A population-based survey of factors relating to the prevalence of falls in older people Gerontology 2005 14 5 340 345 16110237 10.1159/000086372

[B10] Woo J Goggins W Sham A Ho SC Social determinants of frailty Gerontology 2005 14 6 402 408 16299422 10.1159/000088705

[B11] Sulander T Pohjolainen P Karvinen E Self-rated health (SRH) and socioeconomic position (SEP) among urban home-dwelling older adults Arch Gerontol Geriatr 2012 14 1 117 120 21388692 10.1016/j.archger.2011.01.009

[B12] Litwin H Sapir EV Perceived income adequacy among older adults in 12 countries: findings from the survey of health, ageing, and retirement in Europe Gerontologist 2009 14 3 397 406 19386829 10.1093/geront/gnp036 PMC2682171

[B13] Marmot MG Shipley MJ Do socioeconomic differences in mortality persist after retirement? 25 year follow up of civil servants from the first Whitehall study BMJ 1996 14 7066 1177 1180 8916748 10.1136/bmj.313.7066.1177 PMC2352486

[B14] Marmot M Status Syndrome: How Your Social Standing Directly Affects Your Health and Life Expectancy 2004 London: Bloomsbury Publishing

[B15] Lang IA Llewellyn DJ Langa KM Wallace RB Huppert FA Melzer D Neighborhood deprivation, individual socioeconomic status, and cognitive function in older people: analyses from the English Longitudinal Study of Ageing J Am Geriatr Soc 2008 14 2 191 198 18179489 10.1111/j.1532-5415.2007.01557.x PMC2671806

[B16] Lang IA Llewellyn DJ Langa KM Wallace RB Melzer D Neighbourhood deprivation and incident mobility disability in older adults Age Ageing 2008 14 4 403 410 18487260 10.1093/ageing/afn092 PMC2574954

[B17] Blazer DG Social support and mortality in an elderly community population Am J Epidemiol 1982 14 5 684 694 7081200 10.1093/oxfordjournals.aje.a113351

[B18] Seeman TE Lusignolo TM Albert M Berkman L Social relationships, social support, and patterns of cognitive aging in healthy, high-functioning older adults: MacArthur studies of successful aging Health Psychol 2001 14 4 243 255 11515736 10.1037//0278-6133.20.4.243

[B19] Rockwood K Stolee P McDowell I Factors associated with institutionalization of older people in Canada: testing a multifactorial definition of frailty J Am Geriatr Soc 1996 14 5 578 582 8617909 10.1111/j.1532-5415.1996.tb01446.x

[B20] Berkman LF Syme SL Social networks, host resistance, and mortality: a nine-year follow-up study of Alameda County residents Am J Epidemiol 1979 14 2 186 204 425958 10.1093/oxfordjournals.aje.a112674

[B21] Seeman TE Kaplan GA Knudsen L Cohen R Guralnik J Social network ties and mortality among the elderly in the Alameda County Study Am J Epidemiol 1987 14 4 714 723 3631060 10.1093/oxfordjournals.aje.a114711

[B22] Seeman TE Berkman LF Kohout F Lacroix A Glynn R Blazer D Intercommunity variations in the association between social ties and mortality in the elderly. A comparative analysis of three communities Ann Epidemiol 1993 14 4 325 335 8275207 10.1016/1047-2797(93)90058-c

[B23] Fratiglioni L Wang HX Ericsson K Maytan M Winblad B Influence of social network on occurrence of dementia: a community-based longitudinal study Lancet 2000 14 9212 1315 1319 10776744 10.1016/S0140-6736(00)02113-9

[B24] Andrew MK Social capital, health, and care home residence among older adults: A secondary analysis of the Health Survey for England 2000 Eur J Ageing 2005 14 2 137 148 10.1007/s10433-005-0031-8 PMC5547683 28794726

[B25] Kelley-Moore JA Schumacher JG Kahana E Kahana B When do older adults become "disabled"? Social and health antecedents of perceived disability in a panel study of the oldest old J Health Soc Behav 2006 14 2 126 141 16821507 10.1177/002214650604700203 PMC2134789

[B26] St John PD Montgomery PR Cognitive impairment and life satisfaction in older adults Int J Geriatr Psychiatry 2010 14 8 814 821 20623664 10.1002/gps.2422

[B27] Bassuk SS Glass TA Berkman LF Social disengagement and incident cognitive decline in community-dwelling elderly persons Ann Intern Med 1999 14 3 165 173 10428732 10.7326/0003-4819-131-3-199908030-00002

[B28] Mendes De Leon CF Glass TA Berkman LF Social engagement and disability in a community population of older adults: the New Haven EPESE Am J Epidemiol 2003 14 7 633 642 12672683 10.1093/aje/kwg028

[B29] Coleman JS Social capital in the creation of human capital Am J Sociol 1988 14 S95 S120

[B30] Bourdieu P Richardson JG The forms of capital Handbook of Theory and Research for the Sociology of Education 1985 New York: Greenwood 241 258

[B31] Putnam RD Bowling Alone: The Collapse and Revival of American Community 2000 New York: Simon & Schuster

[B32] Putnam RD The Decline of Civil Society: How come? So What? The 1996 John L Manion Lecture 1996 Ottawa: Canadian Centre for Management Development

[B33] Kawachi I Kennedy BP Lochner K Prothrow-Stith D Social capital, income inequality, and mortality Am J Public Health 1997 14 9 1491 1498 9314802 10.2105/ajph.87.9.1491 PMC1380975

[B34] Lochner KA Kawachi I Brennan RT Buka SL Social capital and neighborhood mortality rates in Chicago Soc Sci Med 2003 14 8 1797 1805 12639596 10.1016/s0277-9536(02)00177-6

[B35] Baum F Social capital: is it good for your health? Issues for a public health agenda J Epidemiol Community Health 1999 14 4 195 196 10396543 10.1136/jech.53.4.195 PMC1756864

[B36] Baum F Social capital, economic capital and power: further issues for a public health agenda J Epidemiol Community Health 2000 14 6 409 410 10818114 10.1136/jech.54.6.409 PMC1731698

[B37] Marioni RE Proust-Lima C Amieva H Brayne C Matthews FE Dartigues JF Jacqmin-Gadda H Cognitive lifestyle jointly predicts longitudinal cognitive decline and mortality risk Eur J Epidemiol 2014 14 3 211 219 24577561 10.1007/s10654-014-9881-8 PMC4003346

[B38] Hogan DB MacKnight C Bergman H Models, definitions, and criteria of frailty Aging Clin Exp Res 2003 14 3 Suppl 1 29 14580013

[B39] Grenier A Construction of frailty in the English language, care practice and the lived experience Aging Soc 2007 14 1 21

[B40] Rockwood K Frailty and its definition: a worthy challenge J Am Geriatr Soc 2005 14 6 1069 1070 15935037 10.1111/j.1532-5415.2005.53312.x

[B41] Fried LP Tangen CM Walston J Newman AB Hirsch C Gottdiener J Seeman T Tracy R Kop WJ Burke G McBurnie MA Frailty in older adults: evidence for a phenotype J Gerontol A Biol Sci Med Sci 2001 14 3 M146 156 11253156 10.1093/gerona/56.3.m146

[B42] Andrew MK Mitnitski A Different ways to think about frailty? Am J Med 2008 14 2 e21 18261484 10.1016/j.amjmed.2007.09.017

[B43] Rockwood K Andrew M Mitnitski A A comparison of two approaches to measuring frailty in elderly people J Gerontol 2007 14 7 738 743 10.1093/gerona/62.7.738 17634321

[B44] Mitnitski A Song X Skoog I Broe GA Cox JL Grunfeld E Rockwood K Relative fitness and frailty of elderly men and women in developed countries, in relation to mortality J Am Geriatr Soc 2005 14 2184 2189 16398907 10.1111/j.1532-5415.2005.00506.x

[B45] Mitnitski AB Song X Rockwood K The estimation of relative fitness and frailty in community-dwelling older adults using self-report data J Gerontol 2004 14 6 M627 632 10.1093/gerona/59.6.m627 15215283

[B46] Stokols D Allen J Bellingham RL The social ecology of health promotion: implications for research and practice Am J Health Promot 1996 14 4 247 251 10159704 10.4278/0890-1171-10.4.247

[B47] Bronfenbrenner U The Ecology of Human Development: Experiments by Nature and Design 1979 Boston: Harvard University Press

[B48] Keating N Phillips J Keating N A critical Human Ecology Perspective on Rural Ageing Rural ageing: A good place to grow old? 2008 Bristol: The Policy Press

[B49] Berkman LF Glass T Berkman LF, Kawachi I Social integration, social networks, social support, and health Social Epidemiology 2000 Oxford: Oxford University Press 137 173

[B50] Kawachi I Berkman LF Berkman LF, Kawachi I Social cohesion, social capital, and health Social Epidemiology 2000 Oxford: Oxford University Press 174 190

[B51] Tinetti ME Powell L Fear of falling and low self-efficacy: a case of dependence in elderly persons J Gerontol 1993 14 Spec No 35 38 8409238 10.1093/geronj/48.special_issue.35

[B52] Singh MP Tambay JL Krawchuk S The National Population Health Survey: Design and Issues 1994 Ottawa: Statistics Canada

[B53] National Population Health Survey - Household Component - Longitudinal (NPHS) http://www.statcan.gc.ca/imdb-bmdi/3225-eng.htm

[B54] Woo J Goggins W Sham A Ho SC Public health significance of the frailty index Disabil Rehabil 2006 14 8 515 521 16513584 10.1080/09638280500215867

[B55] Rockwood K Mitnitski A Limits to deficit accumulation in elderly people Mech Ageing Dev 2006 14 5 494 496 16487992 10.1016/j.mad.2006.01.002

[B56] Mitnitski A Fallah N Rockwood MR Rockwood K Transitions in cognitive status in relation to frailty in older adults: a comparison of three frailty measures J Nutr Health Aging 2011 14 10 863 867 22159774 10.1007/s12603-011-0066-9 PMC12878132

[B57] Kulminski AM Ukraintseva SV Culminskaya IV Arbeev KG Land KC Akushevich L Yashin AI Cumulative deficits and physiological indices as predictors of mortality and long life J Gerontol A Biol Sci Med Sci 2008 14 10 1053 1059 18948555 10.1093/gerona/63.10.1053 PMC2684458

[B58] Wilkins R PCCF+ Version 3J User's Guide. Automated geographic coding based on the Statistics Canada Postal Code Conversion Files, including postal codes to May 2002 2002 Ottawa: Health Analysis and Measurement Group, Statistics Canada

[B59] Gonthier D Hotton T Cook C Wilkins R Merging area-level census data with survey data in Statistics Canada Research Data Centres Inform Tech Bull 2006 14 1 21 40

[B60] Data Liberation Initiative http://www.statcan.gc.ca/eng/dli/dli

[B61] Wilkinson RG Unhealthy Societies: The Afflictions of Inequality 1996 London: Routledge

[B62] Lochner K Pamuk E Makuc D Kennedy BP Kawachi I State-level income inequality and individual mortality risk: a prospective, multilevel study Am J Public Health 2001 14 3 385 391 11236402 10.2105/ajph.91.3.385 PMC1446602

[B63] Kulminski AM Ukraintseva SV Kulminskaya IV Arbeev KG Land K Yashin AI Cumulative deficits better characterize susceptibility to death in elderly people than phenotypic frailty: lessons from the Cardiovascular Health Study J Am Geriatr Soc 2008 14 5 898 903 18363679 10.1111/j.1532-5415.2008.01656.x PMC2703425

[B64] Dalgard OS Mykletun A Rognerud M Johansen R Zahl PH Education, sense of mastery and mental health: results from a nationwide health monitoring study in Norway BMC Psychiatry 2007 14 20 17519014 10.1186/1471-244X-7-20 PMC1887526

[B65] Browning C Sims J Kendig H Teshuva K Predictors of physical activity behavior in older community-dwelling adults J Allied Health 2009 14 1 8 17 19361018

[B66] Jang Y Chiriboga DA Lee J Cho S Determinants of a sense of mastery in Korean American elders: a longitudinal assessment Aging Ment Health 2009 14 1 99 105 19197695 10.1080/13607860802154531 PMC2741020

[B67] Lochner K Kawachi I Kennedy BP Social capital: a guide to its measurement Health Place 1999 14 4 259 270 10984580 10.1016/s1353-8292(99)00016-7

[B68] Baum FE Ziersch AM Social capital J Epidemiol Community Health 2003 14 5 320 323 12700212 10.1136/jech.57.5.320 PMC1732452

[B69] Song X Mitnitski A Rockwood K Nontraditional risk factors combine to predict Alzheimer disease and dementia Neurology 2011 14 3 227 234 21753161 10.1212/WNL.0b013e318225c6bc PMC3136058

[B70] Kulminski AM Arbeev KG Ukraintseva SV Culminskaya IV Land K Yashin AI Changes in health status among participants of the Framingham Heart Study from the 1960s to the 1990s: application of an index of cumulative deficits Ann Epidemiol 2008 14 9 696 701 18794010 10.1016/j.annepidem.2008.06.005 PMC2556901

[B71] Cannuscio C Block J Kawachi I Social capital and successful aging: the role of senior housing Ann Intern Med 2003 14 5 Pt 2 395 399 12965964 10.7326/0003-4819-139-5_part_2-200309021-00003

[B72] WHO Global Age-Friendly Cities: A Guide 2007 Geneva: World Health Organization

